# Early changes in cerebral metabolism after perinatal hypoxia-ischemia: a study in normothermic and hypothermic piglets

**DOI:** 10.3389/fped.2023.1167396

**Published:** 2023-05-31

**Authors:** Ted C. K. Andelius, Nikolaj Bøgh, Mette V. Pedersen, Camilla Omann, Mads Andersen, Hannah B. Andersen, Vibeke E. Hjortdal, Michael Pedersen, Martin B. Rasmussen, Kasper J. Kyng, Tine B. Henriksen

**Affiliations:** ^1^ Department of Pediatrics, Aarhus University Hospital, Aarhus, Denmark; ^2^ Department of Clinical Medicine, Faculty of Health, Aarhus University, Aarhus, Denmark; ^3^ The MR Research Centre, Aarhus University, Aarhus, Denmark; ^4^ Department of Cardiothoracic and Vascular Surgery, Aarhus University Hospital, Aarhus, Denmark; ^5^ Comparative Medicine Lab, Aarhus University Hospital, Aarhus, Denmark

**Keywords:** hypoxic-ischemic encephalopathy, animal model, glycerol, cerebral metabolism changes, therapeutic hypothermia

## Abstract

**Introduction:**

Hypoxic ischemic encephalopathy (HIE) after a perinatal insult is a dynamic process that evolves over time. Therapeutic hypothermia (TH) is standard treatment for severe to moderate HIE. There is a lack of evidence on the temporal change and interrelation of the underlying mechanisms that constitute HIE under normal and hypothermic conditions. We aimed to describe early changes in intracerebral metabolism after a hypoxic-ischemic insult in piglets treated with and without TH and in controls.

**Methods:**

Three devices were installed into the left hemisphere of 24 piglets: a probe measuring intracranial pressure, a probe measuring blood flow and oxygen tension, and a microdialysis catheter measuring lactate, glucose, glycerol, and pyruvate. After a standardized hypoxic ischemic insult, the piglets were randomized to either TH or normothermia.

**Results:**

Glycerol, a marker of cell lysis, increased immediately after the insult in both groups. There was a secondary increase in glycerol in normothermic piglets but not in piglets treated with TH. Intracerebral pressure, blood flow, oxygen tension, and extracellular lactate remained stable during the secondary increase in glycerol.

**Conclusion:**

This exploratory study depicted the development of the pathophysiological mechanisms in the hours following a perinatal hypoxic-ischemic insult with and without TH and controls.

## Introduction

Neonatal hypoxic-ischemic encephalopathy (HIE) is a major cause of death and neurodevelopmental impairment in children (1). Brain damage after a perinatal hypoxic-ischemic (HI) insult is a dynamic process that evolves over time (2). During the primary HI insult, an impaired delivery of oxygen and energy substrates to maintain normal cell metabolism will result in an anaerobe metabolism and lactate production (3). Depletion of high-energy substrates, decreased ATP, and Na^+^/K^+^ pump dysfunction will result in a cell depolarization and an influx of calcium, sodium, and water with subsequent cytotoxic edema and cell lysis (4). After the primary HI insult, a period of recovery will follow in which cerebral metabolism is thought to be actively suppressed (5). Despite restored circulation and delivery of oxygen and energy substrates, the HI insult is followed by progressive secondary deterioration with secondary edema, seizures, mitochondrial dysfunction, inflammation, and cell death (6–8). Cell death that occurs after the HI insult is a combination of necrotic and apoptotic cell death and is thought to be time-dependent with necrosis during the early stages followed by apoptosis later on (9, 10). In addition to the neural damage, supportive care is further complicated by multiorgan dysfunction (e.g., cardiovascular dysfunction) (11, 12). Thus, several pathological mechanisms will transpire in the early stages after the HI insult, each occurring at different timepoints and each with the potential for targeted intervention to reduce total damage (2). Therapeutic hypothermia (TH) is the standard neuroprotective treatment for HIE (13). TH has been suggested to ameliorate neural damage by interference with several of the pathophysiological mechanisms that constitutes HIE (6). Preclinical studies have suggested that TH reduces exotoxicity, edema, and inflammation and subsequently inhibits secondary cell death (14–17). TH may further stabilize blood-brain barrier disruption as well as reduce the loss of N-acetylaspartate in the acute phase of injury (18, 19). The pathophysiological mechanisms that constitute HIE have been vigorously investigated, but there is still a lack of evidence regarding the temporal change and interrelation of these mechanisms under normal and hypothermic conditions (3). Basic knowledge on the dynamic of the pathophysiology involved in HIE is key for accurate timing of treatment including development of future potential neuroprotective interventions, prognostication, and monitoring (20). We therefore aimed to map cerebral metabolism, blood flow, oxygenation, and pressure during the first 24 h in piglets subjected to a standardized HI insult with and without TH.

We used a piglet model of HIE, first described in 1996 and later modified by our group (21, 22). The study was approved by and conducted in accordance with the guidelines given by the Danish Animal Experimentation Inspectorate (2016-15-0201-01146) and the Animal Welfare Policy of Aarhus University. We used 24 Danish Landrace piglets, 12–24 h old, with a body weight of 1.2–2.0 kg (Table 1). Animals were provided from herds included in a health-monitoring program for slaughter pigs and were screened for several pathogens that could affect pigs in a production setting. This study is reported according to the animal research: reporting of in vivo experiments (ARRIVE) guidelines (23).

**Table 1:** Demographic and insult severity data for piglets subjected to a hypoxic-ischemic insult (HI) with or without therapeutic hypothermia (HI + TH) and controls.

**Gender (m/f)**	
Control	3/2
HI	4/5
HI + TH	4/4
**Weight (kg)**	
Control	1.50 (1.25–1.70)
HI	1.80 (1.60–1.90)
HI + TH	1.60 (1.50–1.85)
**Time < 7 µV aEEG (min)**	
HI	34.6 (27.7–37.1)
HI + TH	40.5 (23.0–42.2)
**Time MABP < 70% of baseline (min)**	
HI	19.3 (5.0–22.1)
HI + TH	18.0 (12.3–36.3)
**Norepinephrine infusion (µg/kg/min)**	
Control	0/5 (0.00 (0.00–0.00))
HI	**8/9 (0.49 (0.18–0.55))** ^a^
HI + TH	**5/8 (0.45 (0.16–0.69))** ^a^

The number of animals in each group who received norepinephrine infusion is reported. The infusion rate was calculated for each hour and averaged over the 24-h observation period. Data are median with interquartile range. Values with significant in-group differences are bold.

aEEG, amplitude integrated electroencephalography; MABP, mean arterial blood pressure.

^a^ Indicates significance vs. controls.

### Anesthesia and monitoring

Anesthesia was induced with inhalation of 2%–4% sevoflurane. Through peripheral intravenous access, a bolus of propofol (10 mg/kg), fentanyl (30 µg/kg), and rocuronium (1 mg/kg) was administered. The piglet was intubated and mechanically ventilated with a target end-tidal CO_2_ of 4.5–5.5 kPa. Continuous infusion of propofol (4–12 mg/kg/h) and fentanyl (5–10 µg/kg/h) were used as sedation throughout the experiment. Using aseptic techniques, umbilical venous and arterial catheters were placed. Mean arterial blood pressure (MABP), SatO_2_%, FiO_2_, EtCO_2_, temperature, and electrocardiogram (ECG) were monitored and logged (Datex Ohmeda S/5 Collect, Finland). Core temperature was measured continuously approximately 5 cm into the rectum. To prevent infection, all animals were treated with subcutaneous benzylpenicillin 15,000 IE/kg every 12 h or intravenous gentamicin 5 mg/kg once every 24 h and ampicillin 30 mg/kg every 12 h (updated antibiotic regimen mid-trial). A continuous 10% glucose infusion was administered at 5–10 ml/kg/h and continuously adjusted according to blood glucose measurements. Urine production was monitored through regular ultrasound-guided bladder punctures. Blood electrolytes, glucose, and gas composition were regularly monitored. We aimed to maintain a MABP > 40 mmHg. Hypotension was treated initially by reducing infusion of anesthetics. If insufficient, noradrenaline infusion (0.25–1.5 µg/kg/min) and/or dopamine infusion (5–15 µg/kg/min) was titrated to reach MABP > 40 mmHg (24).

### Intracerebral measurements

Using cannulas (2.1 mm diameter for the first and 1.5 mm diameter for the second and third hole), three holes were made through the scalp 10 mm into the brain from the skin surface. The holes were made 10 mm to the left of the midline. The most rostral hole was made 5 mm posterior to a perpendicular line between the eyes, and the two consecutive holes were made in a line 3 mm posteriorly to the first hole and 3 mm apart. In the most rostral hole, a probe for measuring intracranial pressure (ICP) was installed (Codman, Sweden). In the second hole, a probe for measuring cerebral blood flow (CBF) through laser Doppler, temperature, and oxygen tension was installed (Oxford Optronics, UK). In the last hole, a CMA 20 Elite 4 mm membrane microdialysis catheter connected to a CMA 402 syringe pump (CMA Microdialysis, Kista, Sweden) was installed. Microdialysis was performed with central nervous system sterile isotonic perfusion fluid with a flow rate of 0.5 µl/min. The microdialysate was analyzed for lactate, pyruvate, glucose, and glycerol on a CMA 600 microdialysis analyzer (CMA Microdialysis, Kista, Sweden). A cerebral near-infrared spectroscopy (NIRS) oximeter (INVOS 5100, Medtronic, MN, USA) was placed on the parietal region on the right side of the scalp, measuring regional cerebral oxygenation (rSO_2_). A single-channel amplitude-integrated electroencephalogram (aEEG) was acquired with an interelectrode distance of 3 cm and one electrode behind each eye (Natus Medical Incorporated, CA, USA). The aEEG was scrutinized for seizure activity, i.e., rhythmic, evolving activity of a minimum 2 µV and with a duration of >10 s. Due to the supine position of the animal, probes and microdialysis were placed without the use of a stereotactic frame. Standardized device placement was decided based on earlier post-mortem measurements. We have previously investigated the consequence of inserting devices into cerebral tissue and the effect on early measurements in the same animal model (25). Based on these data, animals were left without further interventions for 5 h to allow for the effects of insertion trauma on measurements to resolve.

### Hypoxic-ischemic insult

A global HI insult was induced for 45 min (22). FiO_2_ was reduced to 4%, then adjusted according to aEEG and MABP to ensure survival with a sufficient insult severity. Target was a flat trace aEEG (<7 µV) during the 45-min period combined with hypotension (MABP < 70% of baseline) for at least 10 min. To ensure survival, FiO_2_ was shortly increased if MABP reached <50% of baseline, HR < 80 min^−1^, or aEEG < 3 µV. After 45 min, the piglet was resuscitated with room air. If needed, FiO_2_ was increased to keep SatO_2_ > 90%.

### Experimental protocol

A total of 24 piglets were used. Four piglets were intended as controls without hypoxia or TH. The remaining 20 piglets were subjected to an HI insult and then randomized to normothermia or whole-body TH with a target temperature of 33.5–34.0°C. TH was achieved through passive whole-body cooling by turning of heating blankets and lamps to allow for the cold ambient air in the operation room to cool the piglet to target temperature. TH was continued throughout the experiment and kept stable by the use of alternating the heating blankets and lamps and the cold ambient air. Intracerebral measures were acquired at baseline, during hypoxia, and once every hour for 24 h. After 24 h, the piglets were euthanized with intravenous pentobarbital injection (80 mg/kg). The investigators were not blinded to the group allocation.

### Statistics

A pilot study was performed including three piglets that were healthy and not subjected to HI or TH. Data from these pilot animals showed little variation in intracerebral measurements, and therefore we estimated that four animals were sufficient for the control group. As there was no data on animals with HI and TH, the resource equation method was used to determine the sample size for the two treatment groups (26). With an expected mortality of 20% for piglets exposed to hypoxia, we estimated that 10 animals in each group were sufficient to complete the aim of this study. As the aim was to compare the change in intracerebral measurements, rather than mortality, animal group allocation was adjusted during the study to compensate for eventual mortality in the two HI groups. Data acquired from the Oxford-Optronics probe were recorded in 5–10-min epochs. A mean of those epochs was registered as the hourly value. Due to the expected temporal change in intracerebral measures, the differences between controls, hypothermic animals (HI + TH group), and normothermic animals (HI group) were tested at pre-determined timepoints. Groups were compared at baseline, during hypoxia, at 0–6 h and at 7–24 h. Differences were tested by mixed-effect model analyses with assumed sphericity and randomly missing values, and post-hoc tested using Tukey’s test. Blood-gas values, use of inotropes, and variables describing insult severity were compared by one-way ANOVA or Student's *t*-test for parametric data and Mann–Whitney rank sum test or Kruskal–Wallis test for non-parametric data. The use of inotropes was registered hourly and then averaged for the whole 24-h observation period. The number of animals that received inotropes was reported and for those the average infusion rate was calculated. The change in cerebral perfusion pressure (CPP) was calculated as the difference between MAPB and ICP (27). The relationship between insult severity measured by the duration of EEG suppression, end-hypoxia blood lactate, end-hypoxia blood pH, and end-hypoxia blood standard base excess and glycerol levels were tested by linear regression analysis. A two-sided *p*-value <0.05 was considered statistically significant. Blood-gas values, use of inotropes, and variables describing insult-severity are presented as median with interquartile range (IQR). Intracerebral measurements are presented as mean with standard deviation (SD). The data set generated and analyzed during the current study is available from the corresponding author on request.

## Results

### Insult severity and survival

We were unable to place an umbilical arterial catheter in two animals. As monitoring of arterial blood pressure was required for titration of the HI insult, these two animals were allocated to the control group during preparations. All control and HI + TH animals survived the observation period. Two piglets in the HI group died of refractory hypotension within a few hours after the HI insult. This resulted in nine animals in the HI group, eight in the HI + TH group, and five control animals who survived the whole observation period (Supplementary material S1). The lack of umbilical arterial catheter in the two controls resulted in missing arterial blood-gas samples and blood-pressure readings. Readings failed from two flow probes and one O_2_ probe in the HI group and from one microdialysis catheter in the HI + TH group. aEEG recording during the observation period was impossible in one animal in the control group, one animal in the HI group, and one animal in the HI + TH group due to technical problems. Hypoxia resulted in EEG suppression, metabolic acidosis, and hypotension in both HI-groups (Tables 1, 2). No difference in duration of EEG suppression [34.6 (27.7–37.1) vs. 40.5 (23.0–42.2) min] or hypotension [19.3 (4.9–22.1) vs. 18.0 (12.3–36.3) min] between the HI and HI + TH group was found (Table 1). The degree of metabolic acidosis was similar in the HI and HI + TH group [pH: 6.96 (6.83–7.09) vs. 7.10 (6.95–7.26)]. The average blood glucose concentration was higher in the control group at baseline due to hyperglycemia in one piglet (Table 2). After the insult, blood glucose concentrations were higher in the HI + TH group compared with controls and the HI group and peaked at 12 h (Table 2). The use of norepinephrine was greater in the HI and HI + TH-groups compared with the control group (Table 1). One animal in the HI group received dopamine infusion (average 12.7 µg/kg/min) in addition to norepinephrine.

**Table 2:** Arterial blood-gas values at baseline, during hypoxia, and in recovery in piglets subjected to a hypoxic-ischemic insult with or without therapeutic hypothermia and controls.

	Baseline	Hypoxia		Recovery					
		30 min	45 min	1 h	2 h	4 h	6 h	12 h	24 h
***pH***									
Control	7.44 (7.37–7.51)		7.50 (7.48–7.51)	7.49 (7.47–7.53)	7.46 (7.46–7.50)	7.50 (7.48–7.61)	7.47 (7.47–7.52)	7.46 (7.44–7.47)	7.39 (7.38–7.44)
HI	7.49 (7.44–7.52)	7.07 (7.00–7.27)	**6.96 (6.83–7.09)** ^a^	7.40 (7.26–7.44)	7.48 (7.39–7.54)	7.47 (7.41–7.53)	7.45 (7.41–7.53)	7.44 (7.41–7.49)	7.43 (7.41–7.48)
HI + TH	7.52 (7.48–7.57)	7.18 (7.05–7.27)	**7.10 (6.95–7.26)** ^a^	7.41 (7.33–7.48)	7.49 (7.44–7.55)	7.43 (7.42–7.56)	7.47 (7.44–7.51)	7.43 (7.39–7.44)	7.41 (7.36–7.47)
***pCO2 (kPa)***									
Control	4.65 (4.01–5.30)		4.02 (4.01–4.02)	3.87 (3.85–4.61)	4.07 (4.05–4.86)	3.96 (3.20–4.22)	4.10 (3.81–4.73)	4.56 (4.20–4.77)	4.30 (4.06–4.64)
HI	4.91 (4.41–5.30)	5.60 (3.62–7.16)	6.85 (5.75–7.14)	4.05 (3.70–4.73)	4.21 (3.69–4.45)	4.63 (4.03–5.12)	4.64 (4.08–5.40)	4.91 (4.54–5.28)	4.47 (4.01–5.32)
HI + TH	4.21 (3.52–5.03)	5.99 (4.59–6.65)	6.18 (4.66–6.48)	3.92 (3.53–4.48)	3.88 (3.35–4.56)	4.62 (4.02–5.47)	4.65 (4.20–5.35)	5.47 (4.67–5.80)	5. 95 (4.29–6.29)
***pO2 (kPa)***									
Control	10.29 (8.18–12.40)		10.52 (8.63–12.40)	9.68 (8.54–11.80)	12.60 (8.31–18.70)	11.40 (9.15–12.30)	11.00 (8.95–11.20)	9.58 (8.82–11.78)	11.50 (9.57–12.00)
HI	10.20 (9.08–10.90)	2.63 (2.21–3.37)	4.96 (2.89–13.44)	10.40 (9.61–11.60)	10.10 (8.84–12.15)	10.60 (9.27–11.25)	9.53 (9.29–11.15)	9.39 (9.07–10.60)	10.85 (9.70–13.30)
HI + TH	11.50 (10.11–13.75)	2.78 (2.16–3.19)	**3.86 (3.16–4.74)** ^a^	11.55 (10.30–12.50)	11.95 (10.98–12.35)	12.15 (11.13–13.30)	**13.05 (12.05–14.38)** ^a^ ^,^ ^b^	11.45 (10.40–13.48)	12.60 (11.30–14.60)
***Lactate (mmol/*L*)***									
Control	1.15 (1.10–1.20)		1.30 (1.10–1.50)	1.50 (1.10–1.50)	1.40 (0.90–1.40)	1.80 (1.10–2.00)	1.30 (1.30–1.80)	1.20 (1.03–1.45)	0.90 (0.90–1.80)
HI	1.20 (0.90–1.55)	12.00 (8.90–14.80)	**17.00 (13.95 –20.50)** ^a^	**9.40 (6.20–11.80)** ^a^	2.90 (1.95–6.65)	1.20 (1.00–2.90)	1.25 (1.03–2.03)	1.10 (0.90–1.45)	0.95 (0.90–1.23)
HI + TH	1.00 (0.75–1.50)	11.65 (6.48–13.08)	**10.90 (8.00–17.88)** ^a^	5.15 (2.90–10.35)	2.60 (1.15–4.20)	1.20 (0.85–2.10)	1.20 (0.80–1.58)	1.00 (0.83–1.30)	1.05 (0.83–1.65)
***Base excess (mmol/L)***									
Control	−0.70 (−2.60–1.20)		0.15 (−0.90–1.20)	1.6 (−2.60–3.00)	0.40 (−2.00–2.30)	−0.20 (−0.30–2.60)	0.60 (−1.00–2.10)	−0.15 (−2.08–1.93)	−4.30 (−5.40 – −3.50)
HI	4.40 (1.70–6.00)	−15.50 (−21.15 – −9.60)	**−21.60 (−25.08 – −16.23)** ^a^	−7.70 (−13.60 – −4.10)	−0.02 (−5.40– −1.45)	1.40 (−2.85–3.65)	2.40 (−0.73–5.13)	1.80 (−1.35–3.60)	0.15 (−4.05–3.08)
HI + TH	3.65 (1.88–5.58)	−13.50 (−17.48 – −6.50)	**−14.25 (−24.53 – −8.43)** ^a^	−3.55 (−9.60 – −1.78)	0.20 (−3.73–2.73)	2.30 (−1.83–4.58)	2.80 (−0.13–5.00)	1.55 (−0.38–3.83)	0.55 (−1.95–3.65)
***Glucose (mmol/L)***									
Control	16.95 (7.90–26.00)		16.45 (7.90–25.00)	6.20 (3.70–23.00)	5.80 (3.90–22.80)	7.80 (3.60–21.40)	7.80 (3.40–20.60)	5.15 (3.03–12.15)	6.20 (4.90–7.50)
HI	**5.20 (4.45–6.50)** ^a^	6.60 (5.90–9.60)	10.60 (6.50–13.40)	7.30 (5.00–10.55)	6.00 (4.10–11.35)	5.90 (4.05–10.45)	6.25 (5.25–11.65)	5.20 (4.85–8.35)	5.05 (2.85–6.88)
HI + TH	**6.85 (4.58–8.15)** ^a^	8.85 (6.80–11.30)	11.80 (7.33–15.43)	9.40 (5.60–17.05)	8.30 (6.23–18.33)	7.60 (5.10–20.55)	9.60 (7.20–29.20)	**15.85 (9.70–26.35)** ^b^	**9.95 (7.58–15.00)** ^b^

Data are median with interquartile range. Values with significant in-group differences are bold.

^a^ Indicates significance vs. controls.

^b^ Indicates significance vs. HI or HI + TH.

Seizures were detected in three out of eight animals in the HI group while no seizures were detected in the control and HI + TH group, respectively. No changes in intracerebral-probe measurements, microdialysis-catheter measurements, or NIRS-oximeter measurements were observed in relation to the seizure.

### Vital signs

**Figure 1:**
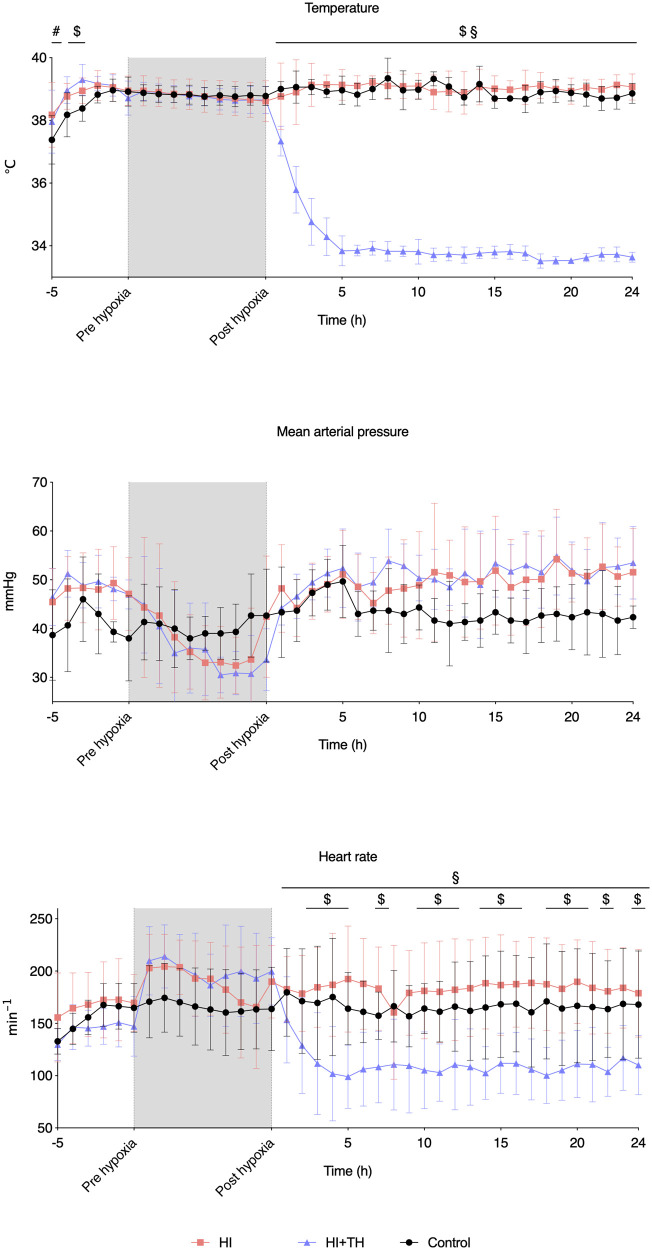
Vital signs at baseline, during hypoxia, and the first 24 h of observation in piglets subjected to a hypoxic-ischemic insult with or without therapeutic hypothermia and controls. Data are mean and standard deviation. #, indicates significance in HI vs. controls; $, indicates significance in HI + TH vs. control; §, indicates significance in HI vs. HI + TH.

After a drop in MABP at the end of hypoxia in both HI groups, MABP remained stable throughout the observation period in all three groups (Figure 1). TH was successfully achieved in all HI + TH animals, and target temperature was reached after approximately 5 h (Figure 1). Hypoxia resulted in initial tachycardia with a subsequent drop toward normal values. Animals treated with TH had a slower heart rate and lower temperature (Figure 1).

### Microdialysis

**Figure 2:**
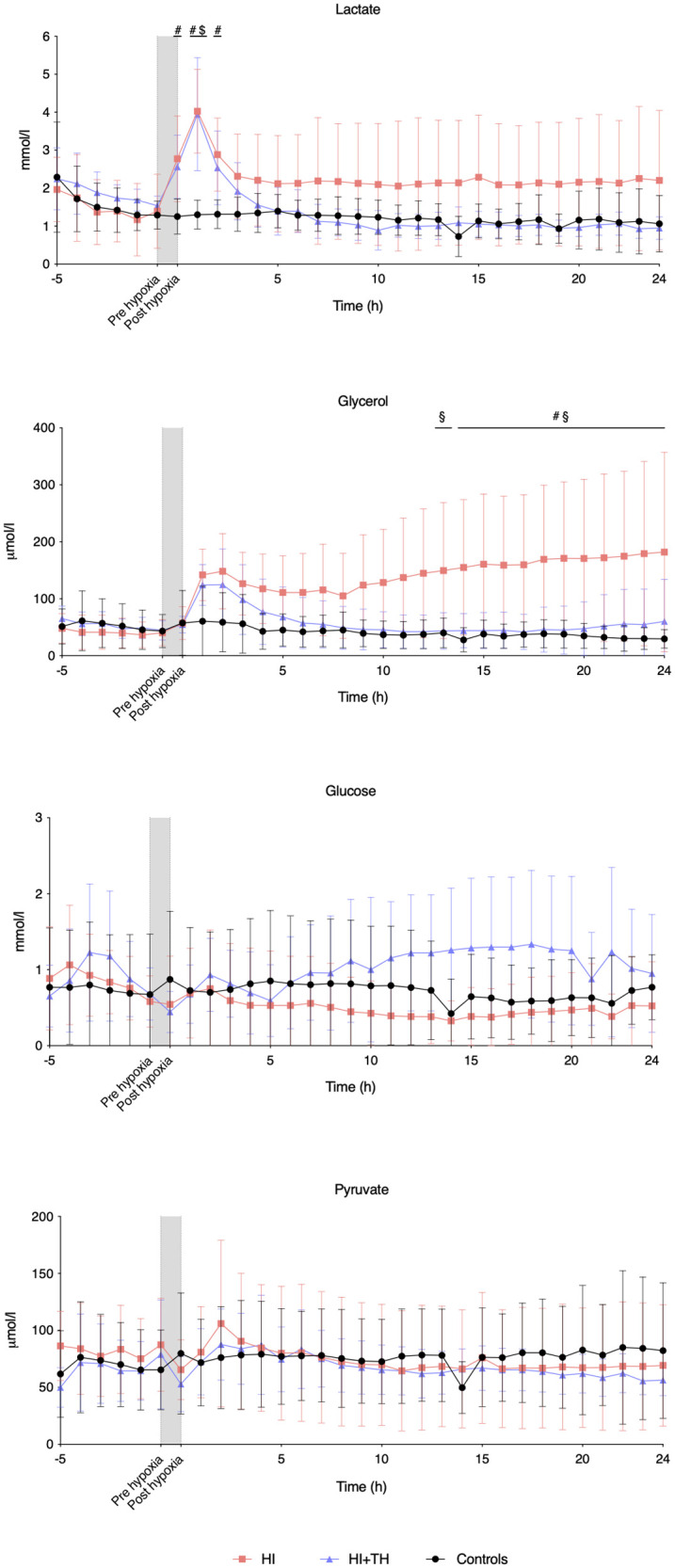
Intracerebral microdialysis data for baseline and the first 24 h in piglets subjected to a hypoxic-ischemic insult with or without therapeutic hypothermia and controls. Data are mean and standard deviation. #, indicates significance in HI vs. controls; $, indicates significance in HI + TH vs. control; §, indicates significance in HI vs. HI + TH.

**Figure 3:**
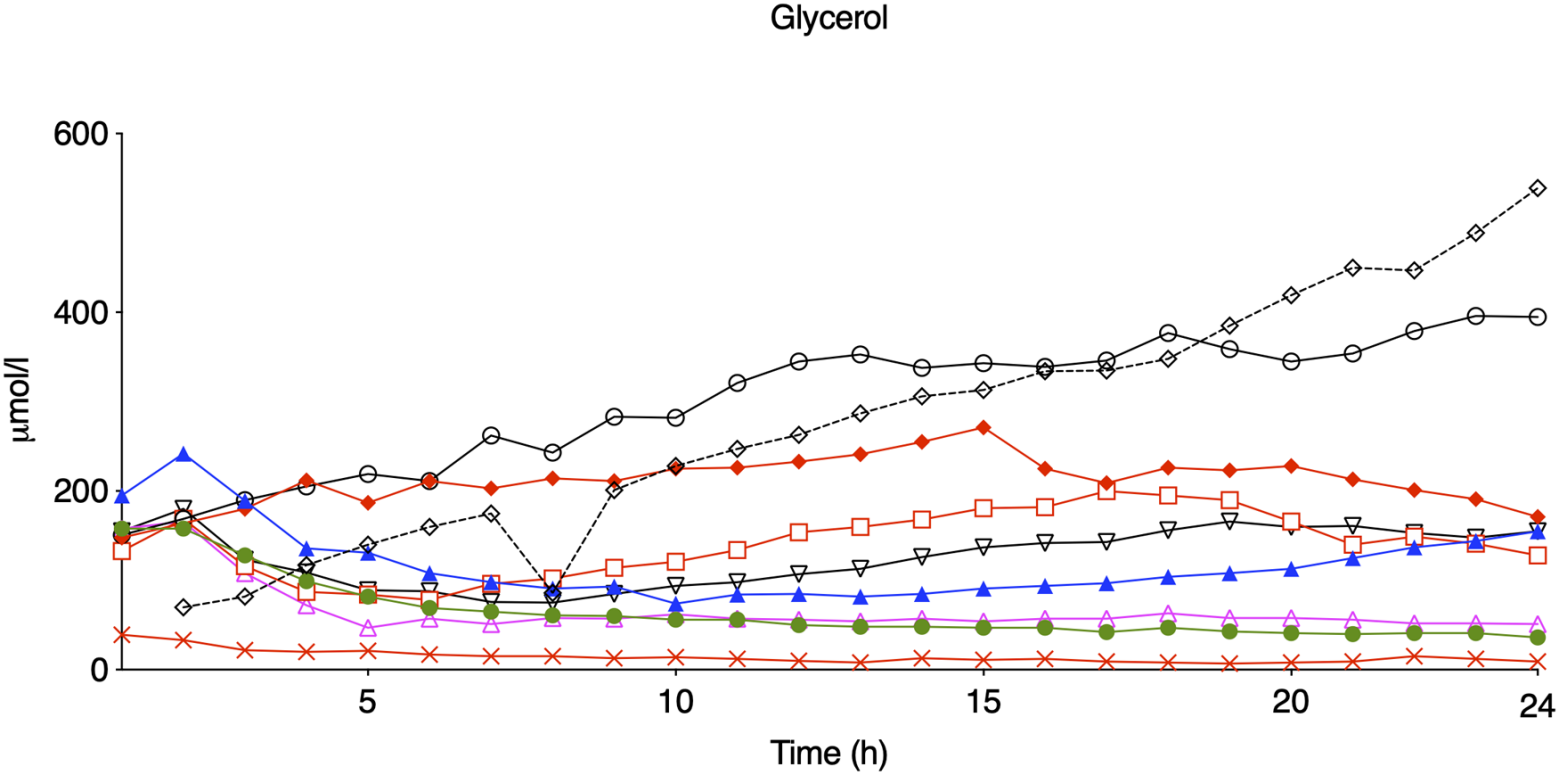
Glycerol levels during the first 24 h of observation in eight piglets subjected to a hypoxic-ischemic insult without treatment with therapeutic hypothermia. Animals who presented with seizures are marked with red.

**Figure 4:**
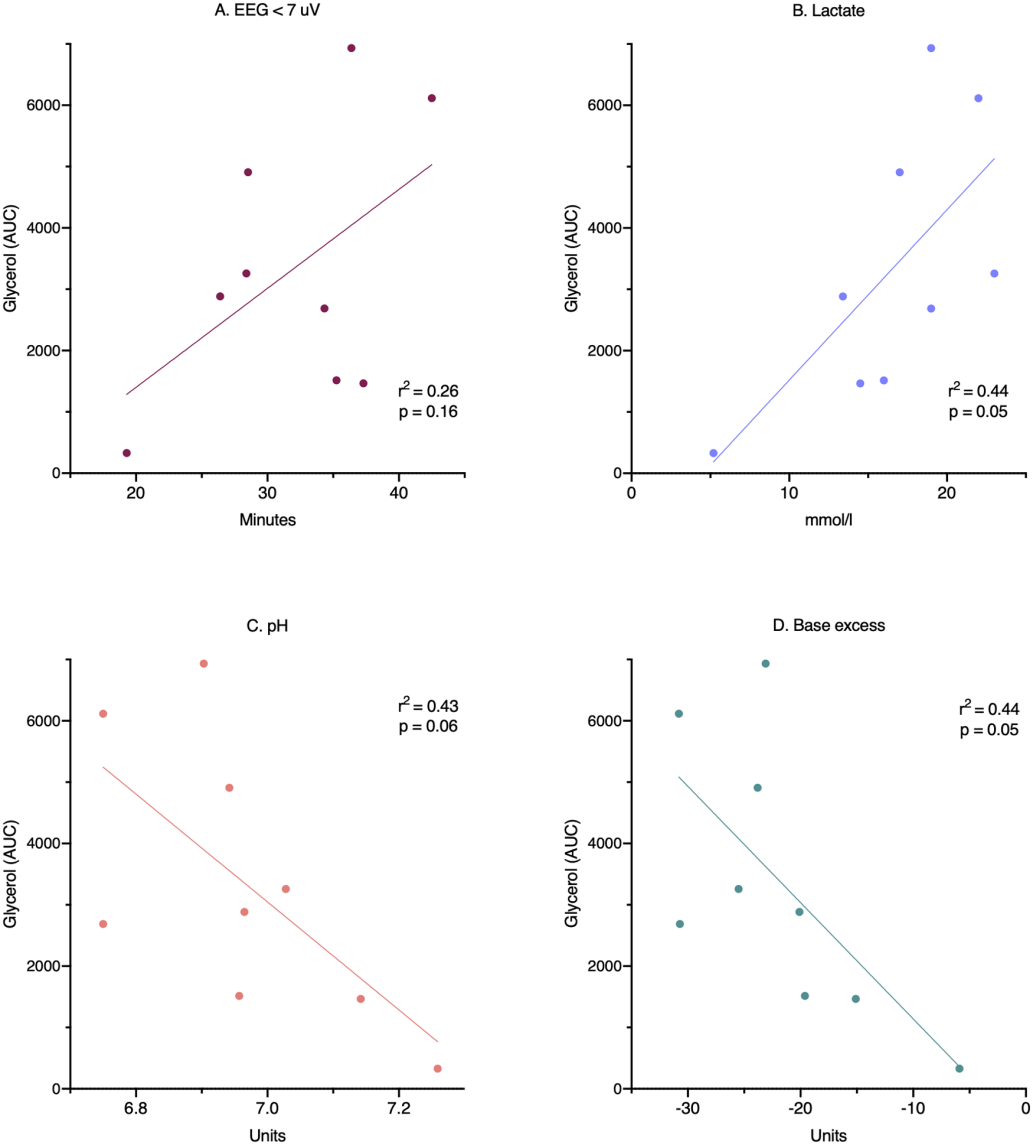
Relationship between insult severity and secondary cell death. Cell death is measured as area under the curve for glycerol during the whole 24-h observation period. Insult severity measured as: (**A**) duration of EEG suppression, (**B**) end-hypoxia blood lactate, (**C**) end-hypoxia blood pH, and (**D**) end-hypoxia blood base excess. AUC, area under the curve; EEG, electroencephalography.

Compared with controls, intracerebral lactate increased immediately after the insult in both groups exposed to HI [controls vs. HI vs. HI + TH (mmol/L); 1.3 (0.4) vs. 4.0 (1.1) vs. 4.0 (1.5)] (Figure 2). There was no difference between the HI and HI + TH group. We found no secondary increase in lactate levels during the 24-h observation period, although lactate levels tended to be higher in the HI group compared with the controls and HI + TH group (Figure 2). Both groups subjected to HI had an increase in glycerol level immediately after the insult [controls vs. HI vs. HI + TH (µmol/L); 60.6 (61.6) vs. 142.1 (45.2) vs. 124.3 (35.7)]. After glycerol levels decreased, a secondary increase in the HI group was observed (Figure 2). From 14 h after the insult, glycerol concentrations were higher compared with those in the control and the HI + TH group and were still increasing at the end of the observation period at 24 h [controls vs. HI vs. HI + TH (µmol/L); 29.6 (16.3) vs. 182.1 (175.3) vs. 60.3 (73.7)] (Figure 2). Six out of nine animals in the HI group presented with a secondary increase in glycerol levels with varying onset, peak, and duration compared with one out of seven animals in the HI + TH group (Figure 3). There tended to be a linear relationship between insult severity, measured by end-hypoxia arterial lactate, pH, standard base excess, and duration of EEG suppression and total increase in glycerol measured as area under the curve (AUC) from end hypoxia to 24 h (Figure 4). There was no overall difference between the three groups with regard to intracerebral glucose and pyruvate (Figure 2). Three animals in the TH + HI group showed an increase in glucose levels during the observation period (Figure 2).

### Intracerebral hemodynamics and rSO_2_

**Figure 5:**
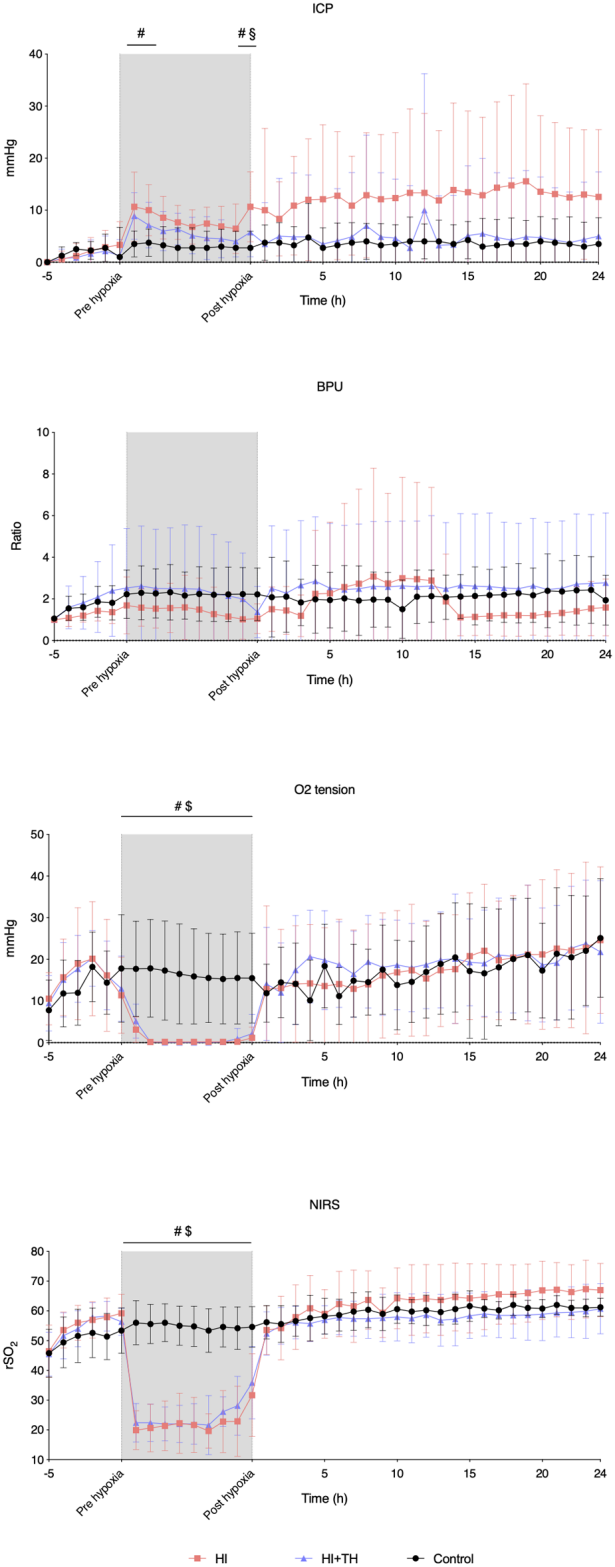
Probe and NIRS data from baseline, during the insult, and for the first 24 h in piglets subjected to a hypoxic-ischemic insult with or without therapeutic hypothermia and controls. Data are mean and standard deviation. #, indicates significance in HI vs. controls; $, indicates significance in HI + TH vs. control; §, indicates significance in HI vs. HI + TH. ICP, intracranial pressure; NIRS, near-infrared spectroscopy; BPU, blood perfusion units; rSO_2_, regional cerebral oxygenation.

The HI insult resulted in an initial increase in ICP in both groups. At the end of the HI insult, animals in the HI group had a higher ICP than animals in the control and HI + TH group. Although not statistically significant, ICP in the HI group seemed to remain higher during the entire observation period. There was no secondary increase in ICP (Figure 5). CBF remained stable during and after the insult in all three groups apart from in one animal in the HI group, which presented with severely increased CBF from the 4th to the 14th hour. CPP tended to decrease during hypoxia, then increased after the insult and remained unchanged during the observation period (Supplementary material S2). Oxygen tension was reduced during the HI insult, increased after reoxygenation, and remained stable in all three groups during the observation period (Figure 5). During the entire observation period, rSO_2_ remained stable in all three groups. Although not statistically significant, rSO_2_ tended to be higher in the HI-group during the observation period. During hypoxia, rSO_2_ was equally suppressed in the HI and HI + TH groups (Figure 5).

## Discussion

This piglet study describes the temporal changes in the pathophysiology evolving over a 24-h period after an HI insult with and without TH treatment and compared with healthy controls.

### Secondary deterioration and glycerol

Glycerol release results from membrane phospholipid degeneration during cell death (28). Membrane phospholipid degeneration is activated through mitochondrial dysfunction and intracellular Ca^2+^ overload mediated by glutamate-activated NMDA receptor activity (29). These processes have been described to mark the onset of secondary deterioration in HIE (6). We found that normothermic animals presented with a secondary increase in glycerol at approximately 14 h after the HI insult with levels still increasing at 24 h. This is in keeping with previous findings of a deteriorating energy state at 24–48 h after the HI insult in normothermic piglets (7). To our knowledge, this pattern has not previously been described by means of a continued direct markers of cell lysis and underlines an important aspect of HIE pathology. Cell death after an HI insult has been shown to consist of a combination of necrosis and apoptosis, possibly occurring at different timepoints and with varying fractions in different parts of the brain (30, 31). Membrane phospholipid degeneration and glycerol production stem from necrosis rather than apoptosis. Total cell death may therefore be more extensive than suggested by glycerol measurements.

Other sources of glycerol production need to be considered. Intracerebral glycerol could originate from breakdown of triglycerides in response to the stress from the HI insult or from altered glycolysis to glycerol 3-phosphate (28, 32). However, the amount of triglycerides in the piglet brain is minimal (33). Secondly, a leak from plasma glycerol produced from the breakdown of triglycerides in adipose tissue could account for part of the acute increase in glycerol but is an unlikely cause for the secondary increase since the blood–brain barrier disruption is only expected to continue for a few hours after the HI insult (34). Lastly, cerebral glucose metabolism to glycerol 3-phosphate is an unlikely source of glycerol as glycolysis to pyruvate and lactate will be favored to optimize ATP production after an HI insult (28).

### Secondary deterioration and lactate

Previous studies based on the same animal model have reported a substantial secondary increase in lactate ranging from 2 h up to 48 h after HI in normothermic piglets measured by magnetic resonance spectroscopy (35, 36). We failed to detect this secondary peak in the continuous microdialysis measurements. Hours after the HI insult, an acute inflammatory reaction will start with subsequent activation and migration of microglia (8, 37, 38). Macrophages are known to have a high intracellular lactate production to meet a high energy demand, which can be measured through MRS (39). As microdialysis only measures extracellular lactate, intracellular microglia-produced lactate will most likely not be detected. Astrocyte-mediated lactate production after an HI insult will be released to the extracellular space through monocarboxylic-acid transporter (MCT)-4 transportation and reabsorbed through MCT-2 transport in the neuron cell membrane (40). The expression of MCT-2 and MCT-4 in neurons and astrocytes have been shown to increase after an HI insult with a peak at 12–24 h in a piglet model of HIE (35). The increased extracellular transportation of lactate may cause the continuously elevated lactate levels observed in our HI group. However, this was not found in the HI + TH group, which had similar lactate levels as the control group. We detected a primary peak in lactate in both groups immediately after the HI insult (Figure 2). This lactate production is possibly caused by the anaerobe metabolism during the HI insult with subsequent leak into the extracellular space through cell lysis. A part of the lactate production could also be caused by the disruption of the blood-brain barrier during the HI insult with subsequent leak of plasma lactate into the cerebral tissue (34).

### Secondary deterioration and cerebral oxygenation, pressure, and blood flow

In accordance with previous studies performed in a similar animal model, we found an instantaneous decrease in oxygen tension and rSO_2_ at the onset of the HI insult (41–43). After the end of the HI insult, oxygen tension and rSO_2_ returned to baseline levels, and we detected no further change during the 24-h observation period. Using NIRS to measure cerebral metabolic rate of oxygen (CMRO_2_) Winter et al. and Tichauer et al. found that CMRO_2_ was decreased in piglets subjected to an HI insult compared with controls hours after the HI insult (44, 45). The decrease in CMRO_2_ occurred in the first hours after the HI insult, and during the same time period, CBF was equal to control animals (45). These findings are in accordance with previously postulated hypothesis of metabolic suppression during the latent phase of injury (5, 46). However, no such change in cerebral metabolism was detected in this study as oxygen tension and rSO_2_ returned to baseline levels after the HI insult. A potential reason for this could be insult severity, i.e., the insult was sufficiently severe to provide a primary and secondary peak in glycerol, but not sufficient to elicit a change in cerebral oxygenation or CBF. Jinnai et al. measured the change in cerebral hemodynamics and oxygenation in piglets subjected to an HI insult similar to ours and found minor or no change in cerebral oxygenation during the first 24 h after the insult (47). They found reduced CBF during the HI insult and during TH treatment, which has also been described by others (41, 42, 47, 48). In contrast, we found CBF to be unchanged during the HI insult despite a tendency to a reduction in CPP and MABP. CBF also remained unchanged during the subsequent treatment with TH. We measured CBF through a device that detects changes in brain microcirculation through a fiber-optic laser Doppler with a tip size ∼450 µm conveyed in the arbitrary unit blood perfusion units. Laser Doppler measures flow in a small area that may not be representative for the total cerebral perfusion. Thus, these results need to be interpreted with caution. However, we have evaluated CBF previously through serial MRI scans with arterial spin labeling during the first 24 h after HI in newborn piglets and found no difference in piglet treated with or without TH (19). In accordance with previous studies in piglets, we found a reduction in HR but stable MABP in TH treated piglets compared with normothermic piglets (49, 50).

A pathophysiological mechanism that constitutes the secondary deterioration after HI is the onset of seizures (6). In our study, seizures were only detected in the HI group in three out of eight animals. Seizures have been shown to elicit changes in cerebral metabolism and hemodynamics in both animal and human neonates detectable through optical monitoring (51, 52). In contrast, we detected no change in oxygen tension, rSO_2_, CBF, or ICP in relation to seizures. In a child with HIE, seizures with a duration less than 7 min did not affect rSO_2_ (53). In a cohort study by Sokoloff et al. with 20 neonates with varying etiologies for seizures, a minor reduction in rSO_2_ of approximately 2%–4% was found during seizures compared with pre-seizure measurements (54). Thus, seizure etiology, duration, or focality may have been too small to influence cerebral metabolism or hemodynamics in our animals. Further, we found no change in microdialysis measurements in relation to seizures. This is in accordance with previous finding in the same animal model (3).

### Secondary deterioration and therapeutic hypothermia

A suggested neuroprotective mechanism of TH is reduction in cerebral metabolism as TH has been shown to reduce metabolism by approximately 5% for each 1-degree reduction in temperature (55). This is supported by our data as piglets in the HI + TH group had lower intracerebral pressure and lower lactate levels, as well as accumulation of intracerebral glucose despite adjustment in peripheral infusion of glucose. The HI + TH group also showed a decrease in heart rate and an accumulation of plasma glucose despite reduction in glucose infusion. Thoresen et al. showed that TH ameliorated secondary energy failure after an HI insult investigated in the new-born piglet (56). This finding is in accordance with our results as the secondary increase in glycerol was absent in the HI + TH group. In a rat model of HIE, Ahn et al. also found that treatment of HIE with TH widened the therapeutic time window for stem-cell treatment (57). As TH reduces metabolism and potentially delays the onset of the pathophysiological mechanisms that constitute the secondary deterioration, early TH treatment may enable other latent phase interventions to be initiated later. Thus, if TH is initiated during neonatal transport, other timely interventions may be effective even if they can only be initiated upon arrival. The reduced metabolism due to TH treatment needs to be taken into consideration when quantifying cerebral damage and metabolism in the acute phase of HIE through other modalities, e.g., magnetic resonance imaging and spectroscopy.

In this study, we found some measurements with borderline significance when comparing the HI with the HI + TH group. A *post hoc* power calculation based on microdialysis and NIRS data showed that the numbers of animals included in each group would need to be more than doubled for sufficient power. We believe that this would contribute with limited information to the manuscript—especially with regard to the 3R principle.

## Conclusion

After a standardized HI insult, we found the presence of a progressive secondary deterioration with concomitant increase in markers of cell lysis, i.e., a secondary increase in glycerol levels in the normothermic piglets. We also found that intracerebral blood flow, pressure, and oxygenation were not influenced during this secondary increase in glycerol levels, and we found no secondary increase in extracellular lactate. TH treatment appeared to abolish the secondary increase in glycerol concentration. TH also tended to result in lower intracerebral pressure, glucose accumulation, and lower levels of extracellular lactate.

## Data Availability

The original contributions presented in the study are included in the article/**Supplementary Material**, further inquiries can be directed to the corresponding author.
