# Supplementary material for: Early changes in cerebral metabolism after perinatal hypoxia-ischemia: a study in normothermic and hypothermic piglets
Source: Front Pediatr. 2023 May 31;11:1167396. doi: 10.3389/fped.2023.1167396 (PMC10264796; doi:10.3389/fped.2023.1167396)
Supplement: Supplementary file 1 [file Table1.docx]

Supplementary Material S1

Article Title

**Early changes in cerebral metabolism after perinatal hypoxia-ischemia; a study in normothermic and hypothermic piglets**

**Ted CK Andelius^1,2*^, Nikolaj Bøgh^3^, Mette V Pedersen^1,2^, Camilla Omann^4^, Mads Andersen^1,2^, Hannah B Andersen^1,2^, Vibeke E Hjortdal^4^, Michael Pedersen^5^, Martin B Rasmussen^1,2^, Kasper J Kyng^1,2^, Tine B Henriksen^1,2^**

^1^ Department of Pediatrics, Aarhus University Hospital, Palle Juul-Jensens Blvd. 99, 8200 Aarhus N, Denmark

^2^ Department of Clinical Medicine, Faculty of Health, Aarhus University, Palle Juul-Jensens Blvd. 99, 8200 Aarhus N, Denmark

^3^ The MR Research Centre, Aarhus University, Palle Juul-Jensens Blvd. 99, 8200 Aarhus N, DK

^4^ Department of Cardiothoracic and Vascular Surgery, Aarhus University Hospital, Palle Juul-Jensens Blvd. 99, 8200 Aarhus N, Denmark

^5^ Comparative Medicine Lab, Aarhus University Hospital, Palle Juul-Jensens Blvd. 99, 8200 Aarhus N, Denmark

**Corresponding author**

Ted Carl Kejlberg Andelius, Department of Pediatrics, Aarhus University Hospital, Palle Juul-Jensens Blvd. 99, 8200 Aarhus N, Denmark. Email; ted.andelius@clin.au.dk. Phone: +45 41637879

## Supplementary Figures

**Supplementary Figure 1.** Flowchart of survival and group allocation.
